# Accessibility of Patients With Special Healthcare Needs to Dental Care in the Eastern Province of Saudi Arabia: A Multicentre Study From Perspective of Caregiver and Dental Care Providers

**DOI:** 10.1155/2024/6905689

**Published:** 2024-09-30

**Authors:** Tarek Ezzeldin, Mazin Algahtani, Nadia Abdulrahman Alghannam, Faisal Abdulkareem Alsafran, Intisar Ahmad Siddiqui, Hebah Zaki Al-Ghanim, Basma Mohamed Bader, Abdullah Abdulatif Alshubat, Adnan Hamad Almarry, Hussein Hejji IbnAhmed, Sarah Abdulhadi Kanakri, Reem Babiker Eltayeb, Mohammed Ibrahim Almuaybid, Khalaf Ali Al-Wasi

**Affiliations:** ^1^Saudi Board Pediatric Dentistry Program, Dammam Specialised Dental Centre, Dammam Medical Complex, Dammam, Saudi Arabia; ^2^Pediatric Dentistry, Department of Preventive Dentistry, College of Dentistry, Imam Abdulrahman Bin Faisal University, Dammam, Saudi Arabia; ^3^Saudi Board Restorative Dentistry Program, Alahsa Dental Centre, Ministry of Health, Alahsa, Saudi Arabia; ^4^Pediatric Dentistry, King Fahad Specialist Hospital, Dammam, Saudi Arabia; ^5^Dental Education, College of Dentistry, Imam Abdulrahman Bin Faisal University, Dammam, Saudi Arabia; ^6^Pediatric Dentistry, Dammam Specialized Dental Centre, Dammam Medical Complex, Dammam, Saudi Arabia; ^7^General Dentistry, Dammam Specialized Dental Centre, Dammam Medical Complex, Dammam, Saudi Arabia; ^8^Pediatric Dentistry, Alahsa Dental Centre, Ministry of Health, Alahsa, Saudi Arabia; ^9^Special Care Dentistry, Dammam Specialized Dental Centre, Dammam Medical Complex, Dammam, Saudi Arabia; ^10^General Dentistry, King Fahad Specialist Hospital, Dammam, Saudi Arabia; ^11^Family Dentistry, Alahsa Dental Centre, Ministry of Health, Alahsa, Saudi Arabia; ^12^Saudi Board Endodontics Program, Dammam Specialized Dental Centre, Dammam Medical Complex, Dammam, Saudi Arabia

**Keywords:** caregiver, dental health, dentist, special healthcare needs, treatment

## Abstract

Dental care accessibility is subject to a dentist's qualification, practice and intention to treat patients, regardless of whether those patients have special healthcare needs (SCN) and should receive treatment in a dental setting. This multicentre study aimed to evaluate the characteristics of patients with SCN, their access to dental care and behaviour towards dental care from their caregiver's perspective. In addition, the perspective of dental care providers who care for patients with SCN and the factors affecting the provision of treatment was also appraised. The Eastern Province of Saudi Arabia served as the site of this cross-sectional study from 1 February 2020 to 31 January 2022. Caregivers of 272 patients with SCN, regardless of age and gender, were recruited in the study. The caregiver's proforma sought information on the demographic characteristics, type of disability, cooperation, medical history, occupation of the parent and patient's behaviour towards oral hygiene and dental healthcare. The second proforma had the dental care provider's perspective about the common disabilities, factors that affect the decision to provide treatment, difficulties patients face in getting their dental treatment and, from the dentist's experience, recommendations to improve the access to dental care for patients with SCN. Statistical analysis was carried out by using SPSS version 22.0. The demographic features, caregiver's perception about oral healthcare accessibility and dental professional's point of view were presented as frequencies and percentages. Chi-square test was applied to compare the proportions. The majority of the caregivers were satisfied with the dental service providers (91.9%) for their patients with SCN. The dental care provider's survey results indicated a shortage of dentists (54.7%) in the region and other factors that pose challenges to special care, like the severity of the disability of patients with SCN (50%), family structure (46.7%), treatment cost (35.6%) and transportation (32.8%). Patients with SCN in Saudi Arabia had a high appraisal of access to dental care and were very satisfied with dental treatment results. However, the presence of a dental care provider in the same rehabilitation centre was a major concern. The severity of the disability and the patient's cooperation were the major factors that may have affected the decision of the dental care provider.

## 1. Introduction

A special healthcare needs (SCN) is defined as “an individual who has a persistent physical, growing, behavioural or expressive disorder, or who is at elevated risk for developing one, and who also needs health and related services that go beyond what is typically needed by children” [1]. Although there are several definitions available in the literature, the majority of them are consistent with the definition provided by the Maternal and Child Health Bureau of the U.S. Department of Health and Human Services, Health Resources and Services Administration [1–5]. In healthcare facilities, the group of patients who either had congenital growth retardation or may develop health impairments in growing age is divided into two broad classifications: first, mental disabilities such as cognitive, behavioural and emotional impairments and second, physical disabilities or disorders, for instance, Tourette syndrome, sickle cell carcinoma, asthma, haemophilia, leukaemia, rheumatic fever, lead toxicities, nephritis and hearing and vision deficits [6].

The degree of difficulty ranges from 25% to 40% in getting access to dental care among patients with SCN in the reported literature, as 35% by Al Agili [3], 25% by Bourke [4], 40% by Burtner and Russel and Kinirons [7]. Patient-to-dentist ratio in Saudi Arabia is 1288:1 dentist [8]. Previous research studies have reported numerous obstacles to dental care for these patients, such as dental fear, low family income, cost of treatment, medical complexity, transportation, a shortage of adequately trained dental providers and inadequate insurance [9–17]. The proficiency and commitment of dental care provider to treat play a significant role in providing timely comprehensive care to patients with SCN. Previous research findings depict that inadequate expertise of dental students of advanced specialized training programmes affected the access of special needs population to dental care [16]. Gaps in the previous studies are as follows:

1. We have little information about how accessible dental care services are to patients with SCN in the Eastern Province.
2. The nature and scope of dental services available to patients with SCN, as well as their degree of satisfaction with the care, are not well documented.
3. No study has examined the features of dental care providers who admit and manage patients with SCN in the Eastern Province.

Around one in seven people worldwide had a disability, which had increased by 1.6% from 2001, according to the findings of the 2006 Participation and Activity Limitation Survey (PALS), which was conducted in Canada. According to a different Canadian Survey on Disability (2012), one in four adults in Canada is classified as having a very severe disability, making up 13.7% of the country's total adult population [2]. In order to investigate the care of children with SCN, Casamassimo, Seale and Ruehs carried out a cross-sectional study from the 2001 National Survey of General Dentists. It was found that just 10% of general dentists had “frequently” or “so often” seen CSHCN [8].

Given the scarcity of wide-age-range studies in the literature, the present study was planned to ensure oral health accessibility, address obstacles and provide necessary guidelines for appropriate dental treatment of the SCN. The objective of this multicentre study was to assess the characteristics of patients with SCN, the accessibility of dental care and the perspectives of caregivers and dental care providers regarding oral healthcare for SCN patients.

## 2. Material and Methods

### 2.1. Description of the Study Area

This cross-sectional multicentre survey-based study was carried out in four centres, i.e., Dammam Specialized Dental Centre of Dammam Medical Complex, King Fahad Specialist Hospital, Al-Hassa Dental Centre (Ministry of Health) and Dental Hospital of Imam Abdulrahman Bin Faisal University. The study lasted 1 year, from 1 February 2021 to 31 January 2022.

### 2.2. Study Design, Sample Size Determination and Sampling

Purposive sampling was used to target a representative sample from a diversified group of participants, in addition to providing an adequate coverage of multicentres in terms of caregivers and dental healthcare providers of SCN patients with complex medical conditions and cognitive or developmental disabilities.

Power analysis was performed to estimate a representative sample size by using the Sample Size Calculator software, by the World Health Organization. Considering the anticipated proportion of caregivers' perceptions of oral health status *p*=56.7% reported by Nqcobo et al. [18], on 6% bond of error, 80% power and 95% confidence range, the minimum representative sample size for caregivers of patients with SCN is 270. While assuming the dentist's knowledge and perception about the oral health status of patients with special needs *p*=60% [19], on a 12.5% margin of error, the required sample size for dental healthcare providers to paediatric patients with special needs is 64 dentists.

The restricted number of specialized clinics for treating patients with SCN nationally, as well as the dentist-patient ratio, contributed to the more than fourfold disparity in sample sizes of caretakers versus dental care providers.

### 2.3. Source Population

Patients with SCN living and dental care providers working in the Eastern Province of Saudi Arabia were the source population.

### 2.4. Study Population

The study population consisted of the household members who were parents or caregivers of patients with SCN.

### 2.5. Inclusion and Exclusion Criteria

The inclusion criteria were followed to recruit the patients with SCN of either gender and age range (7 to 30 years) who were treated at multiple centres (Imam Abdulrahman Bin Faisal University Dental Hospital, Dammam Medical Complex, King Fahad Specialists Hospital-Dammam, King Fahad Hospital, Al Ahsa). The patients with SCN with critical health impairment and high-risk anaesthetic fitness, i.e., American Society of Anaesthesia (ASA) Classes III and IV required for dental treatment, elderly patients (above > 30 years), usually had chronic health impairments, medications, and high risk for anaesthetic and cardiac fitness and those who did not agree to participate in the survey study were excluded. The caregivers of patients with SCN, regardless of age and gender, were invited for the survey.

### 2.6. Study Variables

The study variables were the demographic characteristics of caregivers, overall oral health status, behaviour of patients with SCN towards dental care, consultation, treatment satisfaction and barriers to the accessibility of dental care from the caregiver's point of view and the dentist's perception about the oral healthcare facility, difficulties and recommendations of the dentists from their experience to improve the access to dental care for patients with SCN.

### 2.7. Operational Definitions

Two prevalidated questionnaires used in previous studies [18, 19] were tailored for the study according to the local setting to assess parent caregivers' and dentists' perspectives about the oral healthcare accessibility of patients with SCN. The questionnaires were translated from Arabic to English language by the research team and validated by bilingual experts. The first section of the questionnaire for the parent caregiver requested information on the individual's characteristics, gender, age, nature of disability, medical history, cooperation, oral health condition, dental problems, oral healthcare accessibility and satisfaction of the patients with SCN, as well as the caregiver's demographic characteristics like relationship with patients with SCN and occupation. All items of the questionnaire were closed-ended with additional space to fill specifications in the case of other options (attached as Appendix 1). The second questionnaire had the dentist's perspective about the common disabilities, factors that affect the decision to provide treatment, the difficulties patients face in getting their dental treatment and, from the dentist's experience, recommendations to improve the access to dental care for patients with SCN and a clinical oral examination of oral hygiene (using OHIS index). All items of the questionnaire were closed-ended with additional space to fill specifications in case of other options. Responses to the questions related to the oral health behaviour and dental treatment experience of the patients with SCN from the perspective of the caregiver's perceptions in terms of the one-best subjective answers were assessed. Dentists' perceptions about the dental treatment of patients with SCN, the factors affecting the decision about treatment and difficulties faced by a dentist while treating patients with SCN were also measured in terms of appropriate subjective answers (attached as Appendix 2).

### 2.8. Data Collection and Quality Control

All participants provided written informed consent, and the questionnaire was distributed to them in English or Arabic versions as per their preferences based on nationality. The participants were given 10 min of extra time to complete their questionnaires before they were collected. The respondents' identities were kept anonymous. There were a total of 64 dentists from the Eastern Province, mainly from the same treating facility networks. The study was explained to the participants, and those interested in volunteering were requested to meet in a lecture hall on a predetermined date and time.

### 2.9. Ethics Approval and Consent to Participants

Ethical approval was acquired before conducting the study from the Institutional Review Board (IRB), RAC No. 0610. Consent and authorization for examination were received from the parents as well as from the institutions in order to collect demographic information and oral health status. In the study, a specific proforma was used. The validity of questionnaire was assessed by using Cohen's kappa, a reliability coefficient *k* = 0.621 that revealed high item validity and reliability under Ho: *k* = 0.5.

### 2.10. Data Analysis

SPSS version 22.0, an IBM product designed in Chicago, USA, was used to analyse statistical data. All qualitative variables in our study, including demographic features of caregivers, overall oral health status, behaviour of patients with SCN towards dental care, consultation, treatment satisfaction and barriers for accessibility of dental care from the caregiver's point of view and dentists' perceptions about the oral healthcare facility, difficulties and recommendations of the dentists from their experience to improve the access to dental care for patients with SCN, were presented as frequencies and percentages. Chi-square test was applied to compare the proportions. A *p* value less than or equal to 0.05 was considered a statistically significant result.

## 3. Results

### 3.1. Sociodemographic Characteristics of the Patients With SCN

Among 272 patients with SCN, male preponderance was seen (82.4% vs. 17.6%). The average age of patients was 16.8 ± 5.6 (ranging from 7 to 29 years). The majority of patients (95.2%) were Saudi national, while only 13 (4.8%) were non-Saudi residents. Only 7% of patients had formal education from the school of special education for people with disabilities.

### 3.2. Common Disabilities and Health Impairment of Patients With SCN

**Figure 1:**
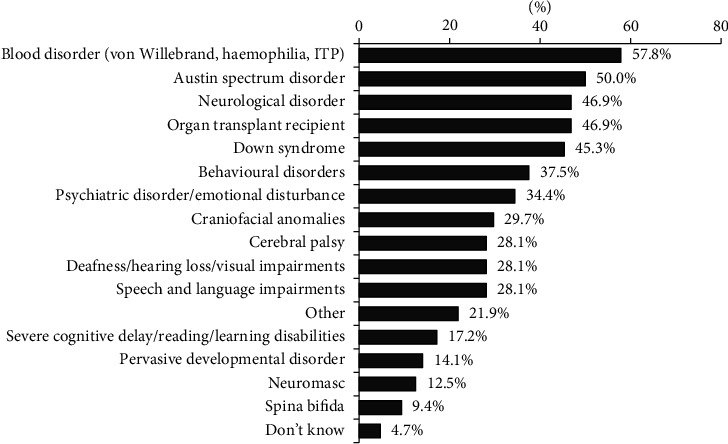
Type of the most common physical disorder and health impairment among patients with special needs presented to the dentist in their practice.

In the patients with SCN, blood disorders were the most common health impairment seen in 57.8% of patients, followed by 50% with autism spectrum disorder, 46.9% with neurological disorders, 46.9% with organ transplants, 45.3% with Down syndrome, 37.5% with behavioural disorders, 34.4% with psychiatric disorders and 29.7% with craniofacial anomalies. There was an equal proportion of patients with SCN with speech and language impairments, cerebral palsy, deafness, hearing impairments and partial or full blindness 28.1%. Eleven (17.2%) patients with SCN had severe cognitive delay, reading and learning disabilities; 14.1% had pervasive development disorder; 12.5% had neuromasc; 9.4% had spina bifida; 4.7% had undefined health impairments; and 21.9% had other noteworthy disabilities, as illustrated in Figure 1.

### 3.3. Sociodemographic Characteristics of the Participants

Of 272 patients with SCN, 26.1% were fathers, 9.2% were mothers, and 64.7% other individuals were caregivers. Although most of the caregivers of patients with SCN were literate up to high school (42.6%) and had a college diploma (37.5%), there were also 18.8% caregivers who did not attend school. Most of the caregivers were either government employees (32.4%) or associated with both governmental and private organizations (37.9%). Sixty-five (23.9%) were also unemployed. The majority of the caregivers had one dependent (59.5%) as detailed in Table 1.

### 3.4. Oral Health Behaviour of the Patients With SCN

The condition of the oral health of patients with SCN according to the caregivers was good in 43%, fair in 39%, and poor in 17.6%, while one (0.4%) caregiver was not sure about the overall tooth condition of his offspring. Many caregivers (56.3%) stated that toothaches occur occasionally in their patients (*p*=0.001), with 19.5% stating that toothaches occur frequently in their patients. Bad oral hygiene was the most common concern (33.1%), followed by toothaches (17.6%), bad mouth smell (17.3%), tooth mobility (4%) and orthodontic treatment needs (4%), and 23.2% of patients had other mixed oral issues. The majority of the patients with SCN (83.8%) were visiting the dentist on a regular basis (*p* < 0.001) as presented in Table 2.

### 3.5. Oral Healthcare and Treatment Experience of the Patients With SCN

When the caregivers inquired about the accessibility of oral healthcare for their patients with SCN, 33.1% of caregivers had no prior experience with oral healthcare, but 27.9% went for examination and screening, 19.1% went for scaling, 11.8% underwent extraction or surgical procedures, 3.7% went through endo-restorative procedures, and 4% attended dental clinics for other dental issues. With regard to the accessibility of oral healthcare for patients with SCN, the majority (93%) received care, 1.8% had cost-related issues, and 0.7% each had face insurance, inconvenient accessibility, dissatisfaction and location-related barriers. None of the patients refused to get an oral health facility. Most of the caregivers of the patients with SCN (91.9%) were very satisfied, and only 1.8% were somewhat unsatisfied. The most significant (*p* < 0.001) oral healthcare perception of the caregivers was the need for a dentist in the centre (56.3%), while 3.3% showed concern about inconvenient appointments and 2.2% were found lacking in other treatment facilities (Table 3).

### 3.6. Sociodemographic Characteristics of the Dentists

Among 64 dentists, significant majority (*p* < 0.001) were specialists of dental care specialities, i.e., 84.4%, and 13.6% were general dental practitioners. Many of the participants (59.4%) worked for the government, while 32.8% worked in universities and 7.8% worked in private or other settings. Most of the participants were practising in Dammam city (79.7%), while 6.3% were from Riyadh and 14.1% were from other parts of the Eastern Province. There was the highest number of paediatric dentists, i.e., 29.7%, followed by 17.2% endodontists and advanced restorative dentists, 7.8% maxillofacial surgeons, 7.8% periodontists, and 21.8% specialist or special care dentists.

### 3.7. Dentist's Perceptions About Factors Affecting the Decision to Treat Patients With SCN

According to the dental treatment provider, the most identified factors that affected the decision to treat patients with SCN were the severity type of disability (75%) as significant (*p* < 0.001) factor, while the patient's cooperation was reported by 50%, the age factor was reported by 32.8%, and some other factors may also affect the decision by 64.1% dentists. From the dentist's point of view, the difficulties patients faced getting their dental treatment were as follows: Shortage of dentists (54.7%) was the significant obstacle (*p* < 0.001), severity type of disability of patients with SCN (50%), family structure (46.7%), treatment cost (35.9%), transport (32.8%), transition (29.7%), insurance (12.5%) and some other reasons (28.1%).

The major recommendations of the dentists from their experience to improve the access of dental care for patients with SCN were the setup of specialized clinics/treatment programmes for special needs (90.6%), community education for oral hygiene (56.3%), availability of special care dentists (32.8%), transition (21.9%) and a few other reasons related to financial support, transport, easy access, priority-based attention and special facilities (Table 4).

## 4. Discussion

### 4.1. Oral Health Accessibility of Patients With SCN From the Perspective of Caregiver and Dentist

This study describes the experiences and challenges that caregivers and dentists face when providing dental care services to patients with SCN in the Eastern Province of Saudi Arabia. The study's findings show that caregivers were aware of the dental care requirements for patients with SCN and that these needs can be met, with the majority of these patients (83.8%) making periodic visits to the dentist. Of those who received dental care, the majority (91.9%) expressed satisfaction. These results contrast with the previous study by Alfaraj et al. [20] who reported that 54.8% of caregivers experience difficulties with accessing dental care in the Eastern Province. This difference in results can be attributed to the changes in the access to medical and dental services due to the COVID-19 pandemic in the Eastern Province. With initial curtailing of diagnostic and treatment visits, the access was subsequently made easier beginning with tele-dentistry and later regular visits along with sedation and general anaesthesia. Along with the accessibility, the changes in the treatment approach by dental service providers also shortened the appointment timings. Usage of atraumatic restorative treatment (ART) protocols and silver diamine fluoride (SDF) was encouraged to treat dental caries. Similar changes in treatment approach have been reported in other studies also [21].

Moreover, the reformed clinical design and environment, including the wheelchair access, made it convenient for the caregiver to get access to the facility.

### 4.2. Dental Treatment Experience and Satisfaction of Patients With SCN

In this study, the majority of patients with SCN (93%) received care, 5 (1.8%) had cost-related issues, and 2 (0.7%) each had face insurance inconvenient accessibility, dissatisfaction and location-related barriers. Despite being challenging for patients with SCN, caregivers and dentists to handle such patients, remarkable majority were taking oral healthcare treatment, while it is known that the Saudi Government Institutions offer free/subsidized services to the patients with SCN. Moreover, the private insurance providers are also regulated to ensure that the costs are affordable by majority.

### 4.3. Caregiver's Viewpoint of Barriers to Accessibility of Oral Healthcare of Patients With SCN

In a recent study [22], the potential barriers to accessibility and routine oral healthcare were reported, with 56% of respondents listing fear towards dental treatment, followed by 52% of patients considering their health impairment was a reason for not seeking dental care and it would increase their fear of dental treatment, and 43% of patients considering physical barriers or the need for carers as barriers to dental care; 10% of people said they had no trouble getting dental care [23].

The majority of the caregivers were satisfied with their dental service providers. Among 64 dentists involved in this study, 54 (84.4%) were specialists, which included 19 paediatric dentists (29.7%), followed by 11 endodontists and advanced restorative dentists (17.2%), 5 maxillofacial surgeons (7.8%), 5 periodontists (7.8%), and 14 specialist or special care dentists (21.8%). This signifies that special care is not just affordable but readily accessible in the Dammam city of Eastern Province.

### 4.4. Dentist's Viewpoint of Barriers to Accessibility of Oral Healthcare of Patients With SCN

The dental care provider's perspective indicated barriers including shortage of dentists, difficulty in treatment of patients with SCN in case of severity type of health impairment or disability, family structure, treatment cost and transportation. There are limited data regarding the experience of dental service providers in treating patients with SCN. Alumran et al. [24, 25] in their studies on prepared and willingness of dental service providers have concluded that compared to females, males seemed more prepared and eager to care for patients with special needs and there are limited data regarding this among the different universities in Saudi Arabia.

In a recent study [26], pre- and postseminar dental student's evaluations of practical courses were taken about their attitude towards treating patients with SCN. The students tended more encouraging ratings than before the start of the programme (*p* < 0.001). However, students' willingness to treat these patients and their social acceptance of them did not substantially alter (*p*=0.681). Hence, it is recommended that dental treatment for patients with SCN be emphasized in the curriculum and focused on in the undergraduate and postgraduate programmes of dental specialities. In general impression from the results of the present study in comparison with reported studies, it revealed that caregivers were well aware of oral health status and accessibility of dental care for their SCN.

## 5. Limitations of Study

The major obstacles encountered include an increased chance of bias because of skewed responses by participants, as the study used a self-administered questionnaire focused on individual impressions of caregivers rather than real existing conditions. Another limitation of the study was that the results in relation to major disabilities and health impairments could not be presented to evaluate the caregiver's and dental care provider's perceptions specific to the particular disabilities due to inadequate cohort sizes based on disabilities and health impairments. The knowledge of caregivers about the oral health status was not evaluated by using some measurement scale from different aspects, but subjective description about overall oral health of patients with SCN was considered in the present study. Future research should explore for more local barriers, evaluate the difficulties found in this study and identify the extent to which they impede access to dental care.

## 6. Recommendations

A one-half of caregivers identified a barrier, the absence of dentists in the same rehabilitation centre for dental treatment instead of transiting to other healthcare centres. It indicated an urgent demand for Special Needs Dental Specialists to benefit the dental clinic in the Eastern Province of Saudi Arabia.

One of the main recommendations derived from the experiences of dental care providers is to train Special Need Dental Specialists and to take steps to raise awareness of oral hygiene among patients with SCN. Hence, it is generally recommended awareness sessions for caregivers about the oral health status and periodic visits of patients with SCN to the dentists to maintain oral hygiene and accessibility of dental care.

## 7. Conclusions

The results of the present study validate the general impression that the access to dental care of patients with SCN and their satisfaction with oral health services were above 90%. The patients with SCN in the Eastern Province of Saudi Arabia had high appraisals of access to care receiving dental care and were very satisfied with dental treatment results. From the dental care provider's side, the major factors affecting the accessibility of dental treatment for patients with SCN were the severity of the disability, the patient's cooperation, their behaviour, the shortage of specialists, their family structure and transport. Hence, this study sets the model for oral health setups and dentists to handle the patient with SCN, as well as to facilitate the underprivileged group.

## Figures and Tables

**Table 1:** Demographic characteristics of caregivers of patients with special healthcare needs.

**Demographic characteristics**	**Categories**	**No. of participants (%)**
Caregiver's relationship with patient	Father	71 (26.1)
	Mother	25 (9.2)
	Others	176 (64.7)⁣^∗^

Education of caregiver	Didn't attend school	51 (18.8)
	High school	116 (42.6)⁣^∗^
	Diploma/college	102 (37.5)
	Higher (Master, Ph.D.)	3 (1.1)

Employment status	Government	88 (32.4)
	Private	16 (5.9)
	Both (Govt./Pvt.)	103 (37.9)
	Unemployed	65 (23.9)

Number of dependents	None	4 (1.5)
	One	162 (59.5)⁣^∗^
	2–3	59 (21.7)
	4 or above	47 (17.3)

⁣^∗^Significantly higher proportion at 5% level of significance.

**Table 2:** Oral health behaviour of patients with special healthcare needs.

**Patient's behaviour**	**Categories**	**No. of participants (%)**
Condition of tooth	Good	117 (43.0)⁣^∗^
	Fair	106 (39.0)
	Poor	48 (17.6)
	Don't know	1 (0.4)

Toothache	Never	63 (23.2)
	Occasionally	153 (56.3)⁣^∗^
	Very often	53 (19.5)
	Don't know	3 (1.1)

Oral health concerns	Can't have oral hygiene	90 (33.1)
	Has teeth pain	48 (17.6)
	Bad mouth smell	47 (17.3)
	Mobile teeth	11 (4.0)
	Needs orthodontic treatment	13 (4.8)
	Others	63 (23.2)

Visit dentist on regular basis	Yes	228 (83.8)⁣^∗^
	No	41 (15.1)
	Don't know	3 (1.1)

⁣^∗^Significantly higher proportion at 5% level of significance.

**Table 3:** Oral health experience of patients with special healthcare needs.

**Oral health experience**	**Categories**	**No. of participants (%)**
Previous dental experience	None	90 (33.1)
	Examination/screening	76 (27.9)
	Scaling	52 (19.1)
	Extraction/surgical	32 (11.8)
	Endo/restorative	10 (3.7)
	Others	11 (4.0)

Oral healthcare accessibility	Received care	253 (93.0)⁣^∗^
	Cost	5 (1.8)
	Insurance	2 (0.7)
	Transport	1 (0.4)
	Inconvenient accessibility	2 (0.7)
	Dissatisfaction	2 (0.7)
	Where to go	2 (0.7)
	Patient refuse	0 (0)
	Treatment ongoing	1 (0.7)
	Preventive treatment	0 (0)
	No referral	11 (4.0)

General satisfaction	Very satisfied	250 (91.9)⁣^∗^
	Somewhat satisfied	17 (6.3)
	Somewhat unsatisfied	5 (1.8)

General oral healthcare perceptions	Needs a dentist in centre	153 (56.3)⁣^∗^
	Inconvenience of appointments	9 (3.3)
	Other treatment facilities	6 (2.2)
	None	104 (38.2)

⁣^∗^Significantly higher proportion at 5% level of significance.

**Table 4:** Dentist's perceptions about dental treatment of patients with SCN.

**Dentist's perception**	**Categories**	**No. of dentists (%)**
Factors affect the decision to treat patients with SCN	Type of severity	48 (75.0)⁣^∗^
	Cooperative	32 (50.0)
	Age	21 (32.8)
	Other^^^	41 (64.1)
	None	1 (1.6)

Difficulties for patients face to get their dental treatment	Behaviour	35 (54.7)⁣^∗^
	Shortage of dentists	35 (54.7)⁣^∗^
	Type of severity	32 (50.0)⁣^∗^
	Family structure	30 (46.7)⁣^∗^
	Financial problem	23 (35.9)
	Transport	21 (32.8)
	Transition	19 (29.7)
	Insurance	8 (12.5)
	Others	18 (28.1)
	None	2 (3.1)

From the dentist's experience, recommendations to improve the access of dental care for patients with SCN	Specialized clinics and treatment programmes for special need	58 (90.6)⁣^∗^
	Community education for oral hygiene	36 (56.3)
	Availability of special care dentist	21 (32.8)
	Transition	14 (21.9)
	Other (financial support, transport, easy access, priority, special facilities)	19 (29.7)

⁣^∗^Significantly higher proportion at 5% level of significance.

^^^Other factors including oral health condition, time for proposed treatment plan, patient's condition to stay for certain time on dental chair, ease for frequent follow-up appointments if required for procedures and medical conditions.

## Data Availability

The survey data are available in the form of original filled papers, Excel spreadsheet and SPSS data sheet which can be provided to the journal as per the requirement (if needed).

## Appendix 1: Questionnaire Regarding the Viewpoint of the SCN Caregiver

  PART ONE: Questions 1 to 4 ask information about the person answering this questionnaire.
1. What is your relationship?
   ○ Mother
   ○ Father
   ○ Stepparent
   ○ Grandparent
   ○ Other (please specify)............
2. Where do you live?
   City ..............
3. What is the highest level of education you have completed?
   ○ Did not attend school
   ○ High school
   ○ Diploma or college
   ○ Higher than college (Master, PhD)
   ○ Prefer not to say
4. What is your profession?
   ○ Government
   ○ Private
   ○ Both government and private
   ○ Unemployed
5. How many dependents do you support?
   ○ _________
  PART TWO: The following questions (5–18) ask information about the young adult recruited to the study.
6. Where does she/he live?
   ○ Same as the respondent.
   ○ Other (please specify)................
7. What is the primary language spoken at his/her home?
   ○ English
   ○ Other (please specify)............
8. How would you describe the condition of her/his teeth and gums?
   ○ Good
   ○ Fair
   ○ Poor
   ○ Don't know
9. How often has she/he experienced toothache/painful gums/sore spots in the past 12 months?
   ○ Never
   ○ Occasionally
   ○ Very often
   ○ Don't know
10. Please tell us any concerns you have about her/his dental health?
   ○ She/he has a bad mouth smell
   ○ She/he has teeth pain
   ○ She/he can't take care of her oral hygiene
   ○ She/he has mobile teeth
   ○ Her/his teeth need orthodontic treatment
   ○ Others (specify)................
11. During the past 12 months, did she/he see a regular/family dentist on a regular basis?
   ○ Yes:
    Dentist's name: ............................ City: ...................
   ○ No
   ○ Don't know
12. Please tell us about her/his previous dental care experiences, if any?
  .......................................................................................................................................................................................................................................................................................................................................................................................................................
13. During the past 12 months, did she/he receive the dental care you felt he/she needed?
   ○ Yes **skip to question number 15**
   ○ No
   ○ Don't know
14. Why did she/he not see a regular/family dentist during the past 12 months OR not get the dental care that she/he needed?
  (Check all that apply)
   ○ Cost too much
   ○ No insurance
   ○ Not available in area/transport problems
   ○ Not convenient times/could not get appointment
   ○ Dentist did not know how to treat or provide care
   ○ Dissatisfaction with dentist
   ○ Did not know where to go for treatment
   ○ Child refused to go
   ○ Treatment is ongoing
   ○ The child does not need any preventive dental treatment
   ○ No referral
   ○ Other (please specify).....................
15. In general, how satisfied are you with her/his access to dental care?
   ○ Very satisfied
   ○ Somewhat satisfied
   ○ Somewhat unsatisfied
   ○ Very unsatisfied
16. Do you want to add anything about her/his oral health OR access to dental care that we missed and you think is important to be mentioned?
  ...........................................................................................................................................................................................................................................................................................................................................................................................................................
  ###

### Appendix 2: Questionnaire Regarding the Viewpoint of the SCN Dental Care Provider

  Questions 1 to 9 ask information about dentists who treat YASHCN
1. What year did you graduate from dental school?
   ○ Year................
2. Did you have any specialty training (including AGD, GPR or hospital training) after dental school:
   ○ No
   ○ Yes (please specify institution) .......................... Year....................
3.  How would rate your dental training in terms of preparing you to treat individuals with special needs?
4. Where is your practice located?
   City .................................
5. What percentage of your patient load is spent treating patients with SHCN?
  ______
6. Please indicate the types of special health care need that you most commonly see in your practice:
  (Check all that apply)
   ○ Autism spectrum disorders (ASDs)
   ○ Bleeding disorders (von Willebrand, hemophilia, ITP)
   ○ Behavioural Disorders (Attention Deficit/Hyperactivity Disorder (AD/HD))
   ○ Pervasive Developmental Disorder
   ○ Cerebral Palsy
   ○ Deafness/Hearing Loss, Visual impairments
   ○ Down Syndrome
   ○ Psychiatric Disorder/Emotional Disturbance
   ○ Neurological disorders (Epilepsy, Traumatic Brain Injury)
   ○ Severe cognitive delay, reading/learning disabilities
   ○ Speech and Language Impairments
   ○ Spina Bifida
   ○ Craniofacial anomalies
   ○ Neuromuscular disorders (Duchenne's muscular dystrophy, ataxia)
   ○ Organ transplant patients (renal, liver, cardiac/lung)
   ○ Other (please specify) ..........
   ○ Don't know
7. Tell us about the factors that affect your decision to treat individuals with special needs? (type and severity of the condition, patient's age, etc)
  .......................................................................................................................................................................................................................................................................................................................................................................................................................................................................................................................................................................................................................................
8. From your experience, what are the difficulties that those patients face to get their dental treatment done? Examples: (*Person-level*: behavior, type and severity of his/her condition. *Family-level*: family structure, financial issues, transportation. *System-level*: insurance coverage, shortage of dentists who accept them, transition issues).
  ..................................................................................................................................................................................................................................................................................................................................................................................................................................................................................................................................................................................................................................................................................................................................................................................................................................................................................................................................................................................................................................................................................................................................................
9. From your experience, what would you recommend to improve the access of patients with special needs to dental care in the eastern province?
  ........................................................................................................................................................................................................................................................................................................................................................................................................................................................................................................................................................................................................................................................................................................................................................................................................................................................................................................................................................................................................................
  ####
